# Networks of ribosome flow models for modeling and analyzing intracellular traffic

**DOI:** 10.1038/s41598-018-37864-1

**Published:** 2019-02-08

**Authors:** Itzik Nanikashvili, Yoram Zarai, Alexander Ovseevich, Tamir Tuller, Michael Margaliot

**Affiliations:** 1 School of Electrical Engineering, Tel-Aviv University, Tel-Aviv, 69978 Israel; 2 Ishlinsky Institute for Problems in Mechanics, Russian Academy of Sciences and the Russian Quantum Center, Moscow, Russia; 3 Sagol School of Neuroscience, Tel-Aviv University, Tel-Aviv, 69978 Israel; 4 Department of Biomedical Engineering, Tel-Aviv University, Tel-Aviv, 69978 Israel

## Abstract

The ribosome flow model with input and output (RFMIO) is a deterministic dynamical system that has been used to study the flow of ribosomes during mRNA translation. The input of the RFMIO controls its initiation rate and the output represents the ribosome exit rate (and thus the protein production rate) at the 3′ end of the mRNA molecule. The RFMIO and its variants encapsulate important properties that are relevant to modeling ribosome flow such as the possible evolution of “traffic jams” and non-homogeneous elongation rates along the mRNA molecule, and can also be used for studying additional intracellular processes such as transcription, transport, and more. Here we consider networks of interconnected RFMIOs as a fundamental tool for modeling, analyzing and re-engineering the complex mechanisms of protein production. In these networks, the output of each RFMIO may be divided, using connection weights, between several inputs of other RFMIOs. We show that under quite general feedback connections the network has two important properties: (1) it admits a unique steady-state and every trajectory converges to this steady-state; and (2) the problem of how to determine the connection weights so that the network steady-state output is maximized is a convex optimization problem. These mathematical properties make these networks highly suitable as models of various phenomena: property (1) means that the behavior is predictable and ordered, and property (2) means that determining the optimal weights is numerically tractable even for large-scale networks. For the specific case of a feed-forward network of RFMIOs we prove an additional useful property, namely, that there exists a spectral representation for the network steady-state, and thus it can be determined without any numerical simulations of the dynamics. We describe the implications of these results to several fundamental biological phenomena and biotechnological objectives.

## Introduction

Gene expression is a complex multistage process in which information encoded in the DNA is used to generate proteins or other gene products. Gene expression involves two primary stages: transcription and translation. Each of these stages involves the sequential movement of enzymes along the genetic material. During transcription, RNA copies of the DNA genes are synthesized by enzymes called RNA polymerase. The product is the messenger RNA (mRNA), which codes, by a series of nucleotide triplets (called codons), the order in which amino-acids need to be combined to synthesize the protein.

Translation is the process in which the information in the mRNA is decoded and the protein is synthesized. During translation, complex macromolecules called ribosomes bind to the start codon in the mRNA and sequentially decode each codon to its corresponding amino-acid that is delivered to the awaiting ribosome by transfer RNA (tRNA). The amino-acid peptide is elongated until the ribosome reaches a stop codon, detaches from the mRNA and the resulting amino-chain peptide is released, folded and becomes a functional protein^1^. The detached ribosome may re-initiate the same mRNA molecule (ribosome recycling^2,3^) or become available to translate other mRNAs. To increase the translation efficiency, multiple ribosomes may decode the same mRNA molecule simultaneously (polysome)^1^.

mRNA translation is a fundamental process in all living cells of all organisms. Thus, a better understanding of its bio-physical properties has numerous potential applications in many scientific disciplines including medicine, systems biology, biotechnology and evolutionary biology. Mechanistic models of translation are essential for: (1) analyzing the flow of ribosomes along the mRNA molecule; (2) integrating and understanding the rapidly increasing experimental findings related to translation and its role in the dynamical regulation of gene expression (see, e.g.^4–12^); and (3) providing a computational testbed for predicting the effects of various manipulations of the genetic machinery. These models describe the dynamics of ribosome flow and include parameters whose values represent the various translation factors that affect the initiation rate and codon decoding times along the mRNA molecule.

Another fundamental biological process based on the flow of biological “machines” along “intracellular roads” is intracellular transport. In this process, vesicles are transferred to particular intracellular locations by molecular motors that haul them along microtubules and actin filaments^1,13^.

We now review two computational models for ribosome flow that are the most relevant for this paper. Numerous other models exist in the literature, see e.g. the survey papers^10,14^.

## Totally Asymmetric Simple Exclusion Process (TASEP)

This is a fundamental model in non-equilibrium statistical mechanics that has been extensively used to model and analyze translation and intracellular transport. TASEP is a discrete-time, stochastic model describing particles hopping along an ordered lattice of *N* sites^15–17^. A particle at site *i* may hop to site *i* + 1 at a rate *γ*_*i*_ but only if this site is empty. This models the fact that the particles have volume and thus cannot overtake one another. Specifically, a particle may hop to the first site at rate *α* (if the first site is empty), and hop out from the last site at rate *β* (if the last site is occupied). In the context of translation, the lattice of sites represents the chain of codons in the mRNA, and the hopping particles represent the moving ribosomes^18,19^.

Analysis of TASEP is in general non trivial, and closed-form results have been obtained mainly for the homogeneous TASEP, i.e. the case where all *γ*_*i*_s are assumed to be equal. The non-homogeneous case is typically studied via extensive and time-consuming Monte Carlo simulations.

In each cell, multiple translation processes take place concurrently, utilizing the limited shared translation resources (i.e. ribosomes and translation factors). For example, a yeast cell contains about 60,000 mRNA molecules and about 240,000 ribosomes^20,21^. The competition for shared resources induces indirect interactions and correlations between the various translation processes. Such interactions must be considered when analyzing the cellular economy of the cell, and also when designing synthetic circuits^22,23^.

Analyzing large-scale translation, as opposed to translation of a single isolated mRNA molecule, is thus an important research direction that is recently attracting considerable research attention^24–29^. For example, in^28^, a TASEP-based computational network consisting of 400 mRNA species and 14,000 ribosomes has been used to analyze the sensitivity of a translation network to perturbations in the initiation and elongation rates and in the mRNA levels. In^29^, a deterministic mean-field approximation of TASEP, called the *ribosome flow model* (RFM), was used for studying the effect of fluctuations in the mRNA levels on translation in a whole cell simulation of an *S*. *cerevisiae* cell.

Here, we consider large-scale networks of interconnected translation processes, whose building blocks are RFMs with suitable inputs and outputs.

## Ribosome Flow Model (RFM)

The RFM^30^ is a continuous-time, deterministic model for ribosome flow that can be obtained via a mean-field approximation of TASEP with open boundary conditions (i.e., the two sides of the TASEP lattice are connected to two particle reservoirs)^31^.

In the RFM, the mRNA molecule is coarse-grained into a chain of *n* consecutive sites of codons. For each site *i* ∈ {1, 2, …, *n*}, a state-variable *x*_*i*_(*t*) ∈ [0, 1] denotes the normalized ribosomal occupancy level (or ribosomal density) at site *i*  at  time *t*, where *x*_*i*_(*t*) = 1 [*x*_*i*_(*t*) = 0] means that site *i* is completely full [empty] at time *t*. The RFM is characterized by *n* + 1 positive parameters: the initiation rate (*λ*_0_), the transition rate from site *i* to site *i* + 1 (*λ*_*i*_), and the exit rate (*λ*_*n*_). Ribosomes that attempt to bind to the first site are constrained by the occupancy level of that site, i.e. the effective flow of ribosomes into the first site is given by *λ*_0_(1 − *x*_1_). This means that: (1) the maximal possible entry rate is *λ*_0_; and (2) the entry rate decreases as the first site becomes fuller, and becomes zero when the first site is completely full. Similarly, the effective flow of ribosomes from site *i* to site *i* + 1 increases [decreases] with the occupancy level at site *i* [*i* + 1] and thus is given by *λ*_*i*_*x*_*i*_(1 − *x*_*i*+1_). This is a “soft” version of the *simple exclusion* principle that models the fact that ribosomes have volume and cannot overtake one another, thus as the occupancy level at site *i* + 1 increases less ribosomes can enter this site, and the effective flow rate from site *i* to site *i* + 1 decreases. The (soft) simple exclusion principle in the RFM allows to model the evolution of ribosomal “traffic jams”. Indeed, if site *i* becomes fuller, i.e. *x*_*i*_ increases then the flow from site *i* − 1 to site *i* decreases and thus site *i* − 1 also becomes fuller, and so on. Recent findings suggest that in many organisms and conditions a non-negligible percentage of ribosomes tends to be involved in such traffic jams (see, for example^32^).

The dynamics of the RFM with *n* sites are given by *n* nonlinear first-order ordinary differential equations (ODEs) describing the change in the occupancy level of each site as a function of time:

1 $$\begin{array}{rcl}{\dot{x}}_{1}(t) & = & {\lambda }_{0}(1-{x}_{1}(t))-{\lambda }_{1}{x}_{1}(t)(1-{x}_{2}(t)),\\ {\dot{x}}_{2}(t) & = & {\lambda }_{1}{x}_{1}(t)(1-{x}_{2}(t))-{\lambda }_{2}{x}_{2}(t)(1-{x}_{3}(t)),\\ {\dot{x}}_{3}(t) & = & {\lambda }_{2}{x}_{2}(t)(1-{x}_{3}(t))-{\lambda }_{3}{x}_{3}(t)(1-{x}_{4}(t)),\\  & \vdots  & \\ {\dot{x}}_{n-1}(t) & = & {\lambda }_{n-2}{x}_{n-2}(t)(1-{x}_{n-1}(t))-{\lambda }_{n-1}{x}_{n-1}(t)(1-{x}_{n}(t)),\\ {\dot{x}}_{n}(t) & = & {\lambda }_{n-1}{x}_{n-1}(t)(1-{x}_{n}(t))-{\lambda }_{n}{x}_{n}(t).\end{array}$$

Note that the *x*_*i*_s are dimensionless, and the *λ*_*i*_s have units of 1/time. The protein *production rate* or *translation rate* is the rate in which ribosomes detach from the mRNA at time *t*, that is, $r(t)\,:\,=\,{\lambda }_{n}{x}_{n}(t)$.

If we let ${x}_{0}(t)\,:\,=\,1$ and ${x}_{n+1}(t)\,:\,=\,0$, then (1) can be written more succinctly as

2 $${\dot{x}}_{i}={h}_{i-1}(x)-{h}_{i}(x),\,i=1,\ldots ,n,$$

where ${h}_{i}(x)\,:\,=\,{\lambda }_{i}{x}_{i}(1-{x}_{i+1})$, and we omit the dependence in time for clarity. This means that the change in the density at site *i* is the flow from site *i* − 1 to site *i* minus the flow from site *i* to site *i* + 1.

Let *x*(*t*, *a*) denote the solution of (1) at time *t* ≥ 0 for the initial condition *x*(0) = *a*. Since the state variables correspond to normalized occupancy levels, we always assume that *a* belongs to the closed *n*-dimensional unit cube denoted [0, 1]^*n*^. Let (0, 1)^*n*^ denote the interior of [0, 1]^*n*^. In other words, $a\in {(0,1)}^{n}$ means that every entry *a*_*i*_ of *a* satisfies 0 < *a*_*i*_ < 1.

It was shown in^33^ that [0, 1]^*n*^ is an invariant set of the dynamics i.e. if $a\in {[0,1]}^{n}$ then $x(t,a)\in {[0,1]}^{n}$ for all *t* ≥ 0. It was also shown that the RFM is a *tridiagonal cooperative dynamical system*^34,35^, and that this implies that (1) admits a steady-state point $e=e({\lambda }_{0},\ldots ,{\lambda }_{n})\in {(0,1)}^{n}$, that is globally asymptotically stable, that is,

$$\mathop{\mathrm{lim}}\limits_{t\to \infty }\,x(t,a)=e,\,{\rm{for}}\,{\rm{any}}\,a\in {[0,1]}^{n}$$

(see also^36^). In particular, the production rate converges to the steady-state value

$${r}_{ss}\,:\,=\,\mathop{\mathrm{lim}}\limits_{t\to \infty }\,r(t)={\lambda }_{n}{e}_{n}.$$

This means that the parameters of the RFM determine a unique steady-state occupancy at all sites along the mRNA. At the steady-state the flow into every site is equal to the flow out of the site. For any initial density the dynamics converge to this steady-state.

### Spectral representation of the RFM steady-state

Simulating the dynamics of large-scale RFMs until (numerical) convergence to the steady-state may be tedious. A useful property of the RFM is that the steady-state can be computed using a spectral approach, that is, based on calculating the eigenvalues and eigenvectors of a suitable matrix^37^. Consider the RFM with dimension *n* and rates *λ*_0_, …, *λ*_*n*_. Define the (*n* + 2) × (*n* + 2) Jacobi matrix

3 $$A({\lambda }_{0},\ldots ,{\lambda }_{n})\,:\,=\,[\begin{array}{ccccccc}0 & {\lambda }_{0}^{-1/2} & 0 & 0 & \ldots  & 0 & 0\\ {\lambda }_{0}^{-1/2} & 0 & {\lambda }_{1}^{-1/2} & 0 & \ldots  & 0 & 0\\ 0 & {\lambda }_{1}^{-1/2} & 0 & {\lambda }_{2}^{-1/2} & \ldots  & 0 & 0\\  &  &  & \vdots  &  &  & \\ 0 & 0 & 0 & \ldots  & {\lambda }_{n-1}^{-1/2} & 0 & {\lambda }_{n}^{-1/2}\\ 0 & 0 & 0 & \ldots  & 0 & {\lambda }_{n}^{-1/2} & 0\end{array}].$$

This is a symmetric matrix, so its eigenvalues are real. Since *A* is componentwise non-negative and irreducible, it admits a unique maximal eigenvalue *σ* > 0 (called the Perron eigenvalue or Perron root), and the corresponding eigenvector $\zeta \in {{\mathbb{R}}}^{n+2}$ (the Perron eigenvector) has positive entries^38^.

#### **Theorem 1**.

*Consider an RFM with dimension n and rates λ*_0_, …,*λ*_*n*_. *Let A be the matrix defined in* (3). *Then the steady*-*state values of the RFM satisfy*^37^:

4 $${r}_{ss}={\sigma }^{-2}\,and\,{e}_{i}={\lambda }_{i}^{-1/2}{\sigma }^{-1}\frac{{\zeta }_{i+2}}{{\zeta }_{i+1}},\,i=1,\ldots ,n.$$

In other words, the steady-state density and production rate in the RFM can be obtained from the Perron eigenvalue and eigenvector of *A*. In particular, this makes it possible to determine *r*_*ss*_ and *e* even for very large chains using efficient and numerically stable algorithms for computing the eigenvalues and eigenvectors of a Jacobi matrix (see, e.g.^39^).

Consider two RFMs: one with rates *λ*_0_, …, *λ*_*n*_ and the second with rates ${\tilde{\lambda }}_{0},\ldots ,{\tilde{\lambda }}_{n}$ such that ${\tilde{\lambda }}_{i}={\lambda }_{i}$ for all *i* except for one index *k* for which ${\tilde{\lambda }}_{k} > {\lambda }_{k}$. Then ${\tilde{\lambda }}_{k}^{-1/2} < {\lambda }_{k}^{-1/2}$. Let *σ* [$\tilde{\sigma }$] denote the Perron root of the matrix $A\,:\,=\,A({\lambda }_{0},\ldots ,{\lambda }_{n})$ [$\tilde{A}\,:\,=\,A({\tilde{\lambda }}_{0},\ldots ,{\tilde{\lambda }}_{n})$]. Comparing the entries of *A* and $\tilde{A}$, it follows from known results in the Perron-Frobenius theory that $\tilde{\sigma } < \sigma$. Hence, ${\tilde{\sigma }}^{-2} > {\sigma }^{-2}$, so Thm. 1 implies that the steady-state production rate in the RFM increases when one (or more) of the rates increases.

Thm. 1 has several more important implications. For example, it implies that *r*_*ss*_ = *r*_*ss*_(*λ*_0_, …, *λ*_*n*_) is a *strictly concave function* on ${{\mathbb{R}}}_{++}^{n+1}$^37^. Also, it implies that the sensitivity of the steady-state with respect to (w.r.t.) a perturbation in the translation rates becomes an eigenvalue sensitivity problem^40^. We refer to the survey^41^ for more details.

Reference^42^ extended the RFM into a single-input single-output (SISO) control system, by defining the production rate as an output, and by introducing a time-varying input $u:{{\mathbb{R}}}_{+}\to {{\mathbb{R}}}_{+}$ representing the flow of ribosomes from the “outside world” into the mRNA molecule. This is referred to as the *RFM with input and output* (RFMIO). The RFMIO dynamics is thus described by:

$$\begin{array}{rcl}{\dot{x}}_{1} & = & u{\lambda }_{0}(1-{x}_{1})-{\lambda }_{1}{x}_{1}(1-{x}_{2}),\\ {\dot{x}}_{2} & = & {\lambda }_{1}{x}_{1}(1-{x}_{2})-{\lambda }_{2}{x}_{2}(1-{x}_{3}),\\  & \vdots  & \\ {\dot{x}}_{n} & = & {\lambda }_{n-1}{x}_{n-1}(1-{x}_{n})-{\lambda }_{n}{x}_{n},\\ y & = & {\lambda }_{n}{x}_{n}.\end{array}$$

**Figure 1:**
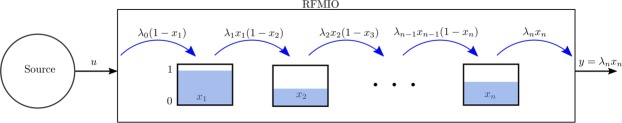
An RFMIO of length *n*, output *y*, and input *u* from an external source.

Note that *u* multiplies *λ*_0_, so that the initiation rate at time *t* is *u*(*t*)*λ*_0_. We consider *λ*_0_ as modeling an intrinsic bio-physical property of the mRNA, and *u*(*t*) as an “outside” effect e.g. the time-varying abundance of “free” ribosomes in the vicinity of the mRNA (see Fig. 1). Throughout, we always assume that *u*(*t*) > 0 for all *t* ≥ 0 in order to avoid some technical problems arising when the initiation rate is zero. Of course, for $u(t)\equiv c$, with *c* > 0, the RFMIO becomes an RFM with initiation rate *cλ*_0_. In this case, the convergence to steady-state represents a form of homeostasis that is sensitive to the value of the input. In particular, the steady-state density of such an RFMIO can be computed using the spectral approach described in Thm. 1.

We write the RFMIO dynamics more succinctly as

5 $$\begin{array}{l}\dot{x}=f(x,u),\\ y={\lambda }_{n}{x}_{n}.\end{array}$$

Let *x*(*t*, *a*, *u*) denote the solution of the RFMIO at time *t* given the initial condition *x*(0) = *a* and input *u*.

The RFMIO facilitates modeling a network of interconnected “roads” (e.g. mRNA or DNA molecules, microtubules, etc.), where the flow out of one intracellular road (representing ribosomes, RNAPs, vesicles attached to molecular motors, etc.) may enter another road in the network or re-enter the same road. This enables the analysis of important phenomena such as translation re-initiation^43^, competition for finite resources including the effect of the exit rate from one road on the initiation rate of other intracellular roads (see e.g.^44^), transport on a network of interconnected microtubules^13^, etc. Such interconnected models are essential for engineering cells for various biotechnological objectives such as optimization of protein production rate, optimization of growth rate, and optimization of traffic jams, as the multiple processes taking place in the cell cannot be analyzed using models of a single “road”^45^.

In the context of translation, the output of an RFMIO represents both the flow of ribosomes out of the mRNA molecule and the synthesis rate of proteins. If the output of a RFMIO is divided into several inputs of other RFMIOs then this represents the distribution of the exiting ribosomes initiating the other mRNAs.

In this paper, we consider networks of interconnected RFMIOs. We show that such dynamical networks provide a useful and versatile modeling tool for many dynamical intracellular traffic phenomena. Our first main result shows that under quite general feedback connections the network admits a unique steady-state and every solution of the dynamics converses to this steady-state. In other words, the network is *globally asymptotically stable* (GAS). This is important for several reasons. For example, GAS implies that the network admits an ordered behavior and paves the way for analyzing further important questions e.g. how does the steady-state depends on various parameters?

We analyze the problem of maximizing the steady-state output of the network. Specifically, the question we consider is how to determine the interconnection weight values between the RFMIOs in the network so that the network output is maximized. Our second main result shows that this is a convex optimization problem, implying that it can be solved using highly efficient algorithms even for very large networks.

In the specific case of feed-forward networks of RFMIOs, we show an additional property, namely, that we can determine the steady-state of the entire network using a spectral approach, and with no need to numerically solve the dynamical equations.

We note that two previous papers considered specific networks of RFMs. Reference^42^ studied the effect of ribosome recycling using a single RFMIO with positive feedback from the output (i.e. production rate) to the input (i.e. initiation rate). Reference^24^ analyzed a closed system composed of a dynamic free pool of ribosomes that feeds a single-layer of parallel RFMIOs. This was used as a tool for analyzing the indirect effect between different mRNA molecules due to competition for a shared resource, namely, the pool of free ribosomes. Here, we study networks that provide a significant generalization of these particular models.

The remainder of this paper is organized as follows. The next section describes the network of RFMIOs that we introduce and analyze in this paper. Then we present our main results and demonstrate them using a biological example. The final section summarizes and describes several directions for future research. To increase the readability of this paper, all the proofs are placed in the Appendix.

We use standard notation. Vectors [matrices] are denoted by small [capital] letters. ${{\mathbb{R}}}^{n}$ is the set of vectors with *n* real coordinates. ${{\mathbb{R}}}_{+}^{n}$ [${{\mathbb{R}}}_{++}^{n}$] is the the set of vectors with *n* real and nonnegative [positive] coordinates. For a (column) vector $x\in {{\mathbb{R}}}^{n}$, *x*_*i*_ is the *i*-th entry of *x*, and *x*′ is the transpose of *x*. If a time-dependent variable *x*(*t*) admits a steady-state then we denote it by *x*_*ss*_, that is, ${x}_{ss}\,:\,=\,{\mathrm{lim}}_{t\to \infty }\,x(t)$.

### Networks of RFMIOs

Consider a network of *m* interconnected RFMIOs. The *input* to the network is a source whose output rate is *y*_0_, and represents external resources that drive the elements in the network. For example, this can represent pools of free ribosomes in the cell. The *output* of the entire network is denoted by *y*. This may represent for example the flow of a desired protein produced by the network, the total flow of ribosomes that feed some other process, etc.

For *i* ∈ {1, …, *m*}, RFMIO *i* is a dynamical system with dimension *n*^*i*^, input *u*^*i*^ and output *y*^*i*^. For any *k* ∈ {0, 1, …, *m*} some or all of the output *y*^*k*^ may be connected to the input of another RFMIO, say, RFMIO *j* with a control parameter (or weight) *v*_*k*,*j*_ ∈ [0, 1]. Here *v*_*k*,*j*_ = 0 means that *y*^*k*^ is not connected to *u*^*j*^. The input to RFMIO *j* is thus ${u}^{j}={\sum }_{k=0}^{m}\,{v}_{k,j}{y}^{k}$.

We define the total network output *y* = *y*^*m*+1^ by

$$y(t)\,:\,=\,\sum _{j=1}^{m}\,{v}_{j,m+1}{y}^{j}(t),$$

where *v*_*j*,*m*+1_ ∈ [0, 1] are the proportion weights from the output *y*^*j*^ of RFMIO *j* to the network output *y*.

We say that the network is *feasible* if: (1) every *v*_*k*,*j*_ ∈ [0, 1]; and (2) ${\sum }_{j=1}^{m+1}\,{v}_{k,j}=1$. The first requirement corresponds to the fact that every *v*_*k*,*j*_ describes the *proportion* of the output *y*_*k*_ that feeds the input of RFMIO *j*. The second requirement means that ${\sum }_{j}\,{v}_{k,j}{y}_{k}={y}_{k}$ i.e. the connections indeed describe a distribution of the output *y*_*k*_ to other points in the network.

We use ${x}_{i}^{j}(t)$, *i* = 1, …, *n*^*j*^, to denote the state-variable describing the occupancy at site *i* in RFMIO *j* at time *t*. The vector

$$z(t)\,:\,=\,[{x}_{1}^{1}(t),\ldots ,{x}_{{n}^{1}}^{1}(t),{x}_{1}^{2}(t),\ldots ,{x}_{{n}^{2}}^{2}(t),\ldots ,{x}_{1}^{m}(t),\ldots ,{x}_{{n}^{m}}^{m}(t){]}^{^{\prime} }\in {[0,1]}^{\ell }$$

aggregates all the state-variables in the network, where $\ell \,:\,=\,{\sum }_{j=1}^{m}\,{n}^{j}$. The variables

$$v\,:\,=\,\{{v}_{k,j}\},\,k\in \{0,1,\ldots ,m\},\,j\in \{1,\ldots ,m+1\}$$

describe the connections between the RFMIOs in the network.

We demonstrate using several examples how networks of RFMIOs, with and without feedback connections, can be used to model and study various intracellular networks. As we will see below, our main theoretical result guarantees that all these networks are GAS. Thus, the state-variables in the networks converge to a steady-state that depends on the various parameters, but not on the initial condition.

Our first example describes the efficiency of ribosome recycling in eukaryotic mRNA, and the tradeoff between recycling on the one-hand and the need to “free” ribosomes for other mRNAs on the other-hand.

#### Example 1.

**Figure 2:**
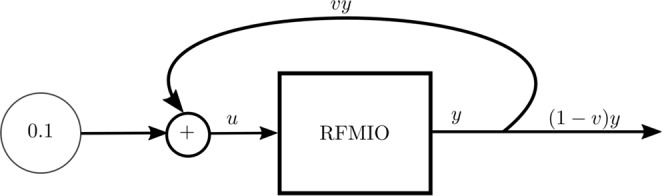
The network in Example 1. The RFMIO contains *n* = 3 sites with rates ${\lambda }_{0}=\cdots ={\lambda }_{3}=2$.

**Figure 3:**
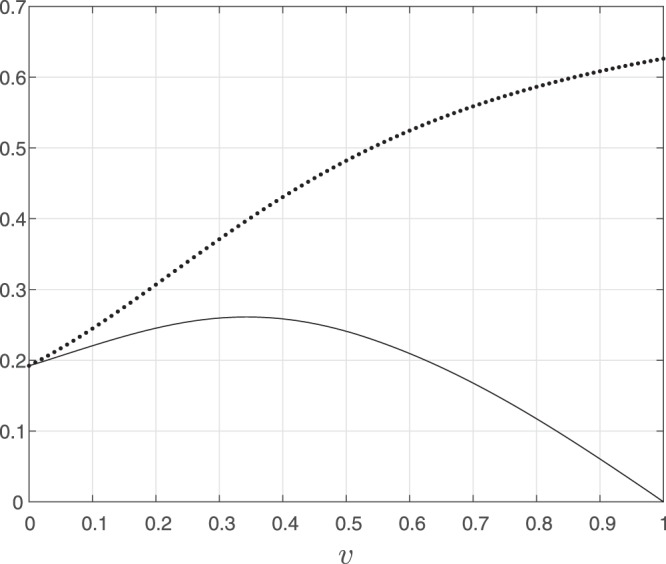
Steady-state values *y*_*ss*_(*v*) (dotted line) and (1 − *v*)*y*_*ss*_(*v*) (solid line) for the system in Example 1 as a function of *v*.

Consider the system depicted in Fig. 2. Here a fixed source with rate 0.1 is feeding an RFMIO of length *n* = 3 with rates ${\lambda }_{0}=\cdots ={\lambda }_{3}=2$. A proportion *v* ∈ [0, 1] of the RFMIO output *y*(*t*) is fed back into the input, so that the total RFMIO input is *u*(*t*) = 0.1 + *vy*(*t*). Figure 3 shows the steady-state values *y*_*ss*_(*v*) and (1 − *v*)*y*_*ss*_(*v*) as a function of *v*. Note that for both these functions there is a unique maximizing value of *v*. We may interpret *y* as the total production rate, and (1 − *v*)*y* as the rate of ribosomes that are *not* recycled, and thus can be used to translate other mRNA molecules. Of course, one can also define other functions as the network output, say, some weighted sum of the production rate and the rate of non-recycled ribosomes. In this case, finding the value *v* that maximizes the steady-state output corresponds to maximizing the production rate on a specific mRNA molecule while still “freeing” enough ribosomes for other purposes.$\square$

Our first result applies to quite general networks of interconnected RFMIOs.

#### **Theorem 2**.

*A feasible network of m RFMIOs admits a globally asymptotically stable steady*-*state point* $e\in {(0,1)}^{\ell }$, *i*.*e*.

$$\mathop{\mathrm{lim}}\limits_{t\to \infty }\,z(t,a)=e,\,for\,all\,a\in {[0,1]}^{\ell }.$$

Theorem 2 implies that all the RFMIO state-variables (and thus the network output) converge to a unique steady-state value. We assume throughout that at steady-state every ${u}_{ss}^{i}$ is positive. Indeed, ${u}_{ss}^{i}=0$ implies that ${y}_{ss}^{i}=0$ and thus RFMIO *i* can simply be deleted from the network.

#### Example 2.

**Figure 4:**
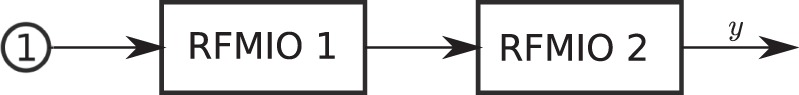
Network of two serially connected RFMIOs in Example 2.

**Figure 5:**
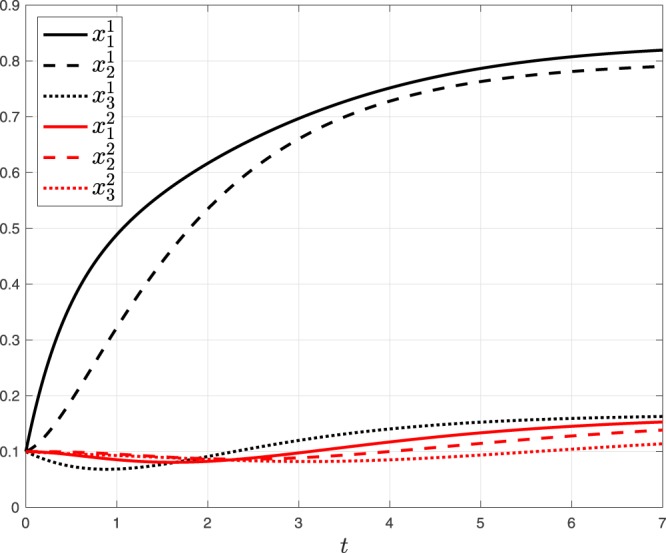
The state variables ${x}_{i}^{1}(t)$ of RFMIO 1 and ${x}_{i}^{2}(t)$ of RFMIO 2, *i* = 1, 2, 3, as a function of *t* for the network in Example 2.

Consider a network with *m* = 2 RFMIOs, where RFMIO 1 is fed with a unit source, and the output of RFMIO 1 feeds the input of RFMIO 2 (see Fig. 4). The output of RFMIO 2 is defined as the network output *y*. Both RFMIOs have dimension *n* = 3, RFMIO 1 with rates $[{\lambda }_{0}^{1},{\lambda }_{1}^{1},{\lambda }_{2}^{1},{\lambda }_{3}^{1}]=[1,1,1/4,1]$, and RFMIO 2 with rates [1, 1, 1, 1]. Figure 5 depicts the state-variables ${x}_{i}^{1}(t)$ of RFMIO 1 and ${x}_{i}^{2}(t)$ of RFMIO 2, *i* = 1, 2, 3, as a function of *t*, for the initial condition ${x}_{i}^{1}(0)={x}_{i}^{2}(0)=1/10$. Note that the rate *λ*_2_ = 1/4 in RFMIO 1 leads to a “traffic jam” of ribosomes in this RFMIO, that is, the steady-state densities in the first two sites are high, whereas the density in the third site is low. This yields a low output rate from this RFMIO. The second RFMIO thus converges to a steady-state with low densities.

Example 2 may represents a case of re-initiation: one ORF appears in the 5′UTR of the second ORF and the ribosomes finishing the translation of the first ORF start translating the second one^43,46,47^. In this case, a low elongation rate along the first ORF is expected to yield a low density of ribosomes in the second ORF.

## Optimizing the Network Output Rate

Several papers considered optimizing the production rate in a *single* RFM or some variant of the RFM^37,48–51^. Here, we study a different problem, namely, maximizing the steady-state output in a network of RFMIOs w.r.t. the control (or connection) weights in the network. In other words, the problem is how to distribute the traffic between the different RFMIOs in the network so that the steady-state output is maximized.

### **Problem 1**.

Given a network of *m* RFMIOs with a network output

$$y(t)\,:\,=\,\sum _{j=1}^{m}\,{v}_{j,m+1}{y}^{j}(t),$$

maximize the steady-state network output ${y}_{ss}\,:\,=\,{\mathrm{lim}}_{t\to \infty }\,y(t)$ w.r.t. the control variables *v*_*k*,*j*_ subject to the constraints:

6 $$\begin{array}{lll}{v}_{k,j} & \in  & [0,1]\,{\rm{for}}\,{\rm{all}}\,k\in \{0,1,\ldots ,m\},\,j\in \{1,\ldots ,m+1\},\\ \sum _{j}\,{v}_{k,j} & = & 1\,{\rm{for}}\,{\rm{all}}\,k\in \{0,1,\ldots ,m\}.\end{array}$$

The constraints here guarantee the feasibly of the network. However, the results below remain valid even if the second constraint in (6) is replaced by ${\sum }_{j}\,{v}_{k,j}\le 1$ for all *k* ∈ {0, 1, …, *m*}.

The next result is instrumental for analyzing Problem 1.

### **Proposition 1**.

*Under a suitable reparametrization Problem 1 becomes a convex optimization problem*.

The following example demonstrates this result.

### Example 3.

Consider an RFMIO with a single site and rates *λ*_0_ = *λ*_1_ = 1:

7 $$\begin{array}{rcl}{\dot{x}}_{1} & = & (1-{x}_{1})u-{x}_{1},\\ y & = & {x}_{1}.\end{array}$$

Suppose that the input is *u* = 0.1 + *vy*, where *v* ∈ [0, 1], that is, there is a feedback connection from the output of the RFMIO back to the input with a weight *v*. It is straightforward to verify that for any *x*_1_(0) ∈ [0, 1] the solution *x*_1_(*t*) converges to the value

$${e}_{1}(v)\,:\,=\,\{\begin{array}{cc}\tfrac{v-1.1+\sqrt{{(1.1-v)}^{2}+0.4v}}{2v} & {\rm{if}}\,v > 0,\\ 1/11 & {\rm{if}}\,v=0.\end{array}$$

It is also straightforward to verify that $\frac{{d}^{2}}{d{v}^{2}}{e}_{1} > 0$ for all *v* ∈ (0, 1), so *e*_1_ and thus the steady-state output *y*_*ss*_(*v*) = *e*_1_(*v*) is *not* concave in *v*. We conclude that the optimization problem:

8 $${\rm{\max }}\,{y}_{ss}(v)\,{\rm{subject}}\,{\rm{to}}\,v\in [0,1]$$

is *not* a convex optimization problem. Nevertheless, since in this particular case *y*_*ss*_(*v*) is a scalar function, it is easy to solve this optimization problem yielding (all numerical values in this paper are to four digit accuracy)

9 $${y}_{ss}^{\ast }\,:\,=\,{y}_{ss}(1)=\mathrm{0.2702.}$$

The reparametrization is based on redefining the input as *u* = 0.1 + *w*, with the constraint *w* ∈ [0, *y*]. Now the steady-state output of (7) is ${y}_{ss}(w)=\frac{0.1+w}{1.1+w}$, and this function is strictly concave in *w*. At steady-state, the constraint *w* ≤ *y* means that $w\le \frac{0.1+w}{1.1+w}$. Thus, now the maximization problem is

10 $${\rm{\max }}\,{y}_{ss}(w)\,{\rm{subject}}\,{\rm{to}}\,0\le w\le \frac{0.1+w}{1.1+w},$$

and this constraint defines a convex set of admissible *w*’s, so (10) is a convex optimization problem. The solution of this problem is obtained at w* = 0.2702 for which *y*_*ss*_(w*) = 0.2702. We conclude that the optimal values correspond to ${w}^{\ast }={y}_{ss}^{\ast }$, and this implies that the solution to the optimization problem (8) is v* = 1. Thus, we can obtain the optimal weights from the solution of the reparametrized problem.$\square$

The next example demonstrates a synthetic and more complex network that includes feedback connections.

### Example 4.

**Figure 6:**
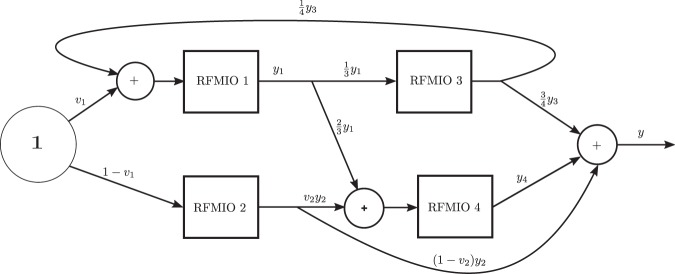
Topology of the network in Example 4.

**Figure 7:**
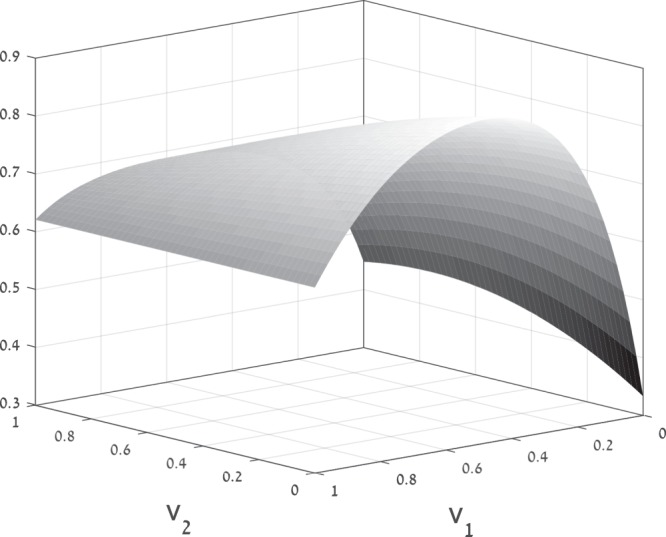
Steady-state output *y*_*ss*_(*v*_1_, *v*_2_) for the network in Example 4 as a function of *v*_1_, *v*_2_.

Consider the network depicted in Fig. 6. The network consists of four RFMIOs. RFMIO 1 and RFMIO 4 have dimension *n* = 4, and rates [2, 2, 2, 2, 2]. RFMIO 2 and RFMIO 3 have dimension *n* = 3, and rates [1, 1, 1, 1]. A unit source feeds RFMIO 1 and RFMIO 2 with proportions *v*_1_ and 1 − *v*_1_, respectively. Another control parameter, *v*_2_, determines the division of the output of RFMIO 2. The total network output is defined as $y:\,=\frac{3}{4}{y}_{3}+{y}_{4}+(1-{v}_{2}){y}_{2}$. Figure 7 depicts the steady-state output as a function of the control parameters *v*_1_, *v*_2_. It may be seen that *y* is a concave function. The optimal output value y* = 0.8595 is obtained for ${v}_{1}^{\ast }=0.48$, and ${v}_{2}^{\ast }=0$. The value ${v}_{2}^{\ast }=0$ is reasonable, as this implies that all the output *y*_2_ of RFMIO 2 goes directly to the network output *y* rather than first to RFMIO 4 and from there, indirectly, to *y*.$\square$

**Figure 8:**
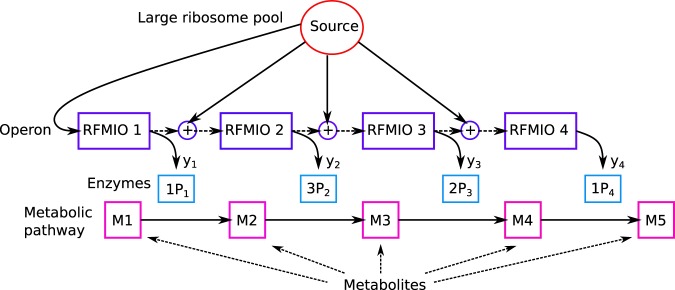
A network describing an operon translation model and a corresponding metabolic pathway.

To further explain the biological motivation of the optimization problem studied here, consider for example the metabolic pathway or protein complex described in Fig. 8. This includes a set of enzymes/proteins that are involved in a specific stoichiometry (see, for example^12^). In this case the objective function is of the form *b*′*y*, where *y* is a vector of production rates of the different proteins in the metabolic pathway or protein complex, and *b* is their stoichiometry vector. Figure 8 depicts an operon with four coding regions on the same transcript. The initiation rate to each ORF is affected by an “external” factor (e.g. the intracellular pool of ribosomes), and also by the “leakage” of ribosomes from the previous ORF. The proteins produced in the operon (*P*_1_, …, *P*_4_) with production rates *y*_1_, …, *y*_4_ are part of a metabolic pathway where they are “needed” with a stoichiometry vector *b* = [1, 3, 2, 1]′.

### Analysis of feed-forward networks

In this section we further analyze *feed*-*forward* networks of RFMIOs, where feed-forward means that for any *j* the input *u*_*j*_ of RFMIO *j* does not depend either directly or indirectly on the output *y*_*j*_ of RFMIO *j*. In other words, there are no feedback connections. In terms of graph theory, this means that the graph describing the connections is a directed acyclic graph (DAG). For these networks Problem 1 can be solved in a more direct way.

#### **Proposition 2**.

*Consider a feed*-*forward network of RFMIOs*. *Let v denote the collection of all control weights*. *The mapping v* → *y*_*ss*_(*v*) *is strictly concave*.

The following example demonstrates this result.

#### Example 5.

**Figure 9:**
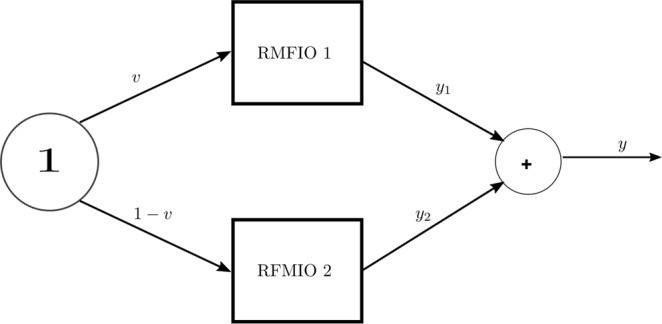
Two RFMIOs fed from a common unit source. The input to RFMIO 1 is ${u}_{1}(t)\equiv v\in [0,1]$, and the input to RFMIO 2 is ${u}_{2}(t)\equiv 1-v$. The network output is defined as the sum of the RFMIO outputs $y(t)\,:\,=\,{y}_{1}(t)+{y}_{2}(t)$.

**Figure 10:**
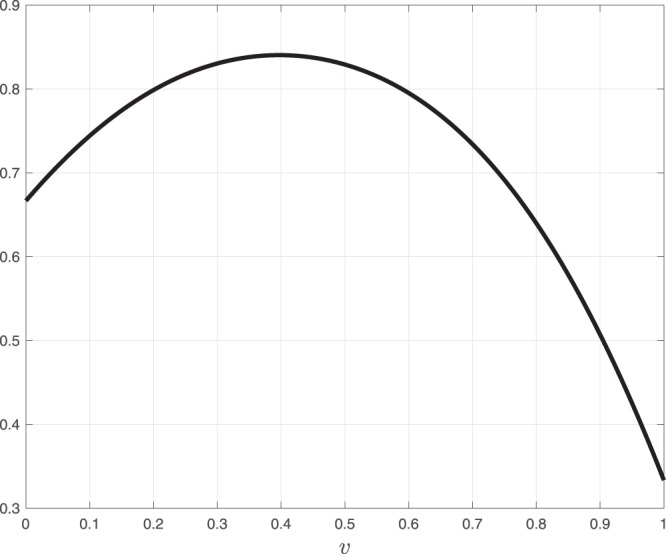
Steady-state network output *y*_*ss*_(*v*) for the network in Example 5 as a function of the control parameter *v*.

Consider the network of two RFMIOs described in Fig. 9. Each RFMIO has dimension *n* = 3. The rates of RFMIO 1 are $[{\lambda }_{0}^{1},{\lambda }_{1}^{1},{\lambda }_{2}^{1},{\lambda }_{3}^{1}]=[1,1,1,1]$, and those of RFMIO 2 are [2, 2, 2, 2]. In other words, every rate in RFMIO 1 is slower than the corresponding rate in RFMIO 2. A unit source feeds both RFMIO 1 with *u*_1_ = *v*, and RFMIO 2 with *u*_2_ = 1 − *v*. The network output *y*(*t*) is defined to be the sum of the two RFMIO outputs. Figure 10 depicts the network steady-state output as a function of *v* ∈ [0, 1]. It may be seen that *y*_*ss*_(*v*) is a strictly concave function of *v*, and in particular that there exists a unique value v* = 0.3971 for which *y*_*ss*_ is maximized. This corresponds to feeding a smaller [larger] part of the joint source to RFMIO 1 [RFMIO 2]. This is reasonable, as RFMIO 1 has slower rates than RFMIO 2. Hence, it is possible to “direct” more traffic to RFMIO 2 while still avoiding “traffic jams” in this RFMIO.$\square$

This example demonstrates the problem of dividing a common resource in a biological network between several “clients” such that some overall performance measure is optimized. For example, this network can represent the problem of optimizing heterologous protein levels by introducing a copy of the gene to the host genome in *multiple* locations. This raises the question of the optimal strength of the initiation rate of the different gene copies (engineered via manipulation of the 5′UTR and the beginning of the ORF, e.g. via the introduction of Shine-Dalgarno sequence with different strengths and manipulation of the mRNA folding in this region)^52,53^.

## Spectral Representation of the Network Steady-State

Recall that in a single RFM it is possible to obtain the steady-state density (and thus the steady-state production rate) using a spectral approach and without numerically simulating the dynamics. The same property immediately carries over to feed-forward networks of RFMIOs. To explain this, consider an RFMIO, say RFMIO *j*, that is fed only by a constant source. This is just an RFM and its steady-state density and output can be calculated as in Thm. 1. Now consider an RFMIO that is fed by the output of RFMIO *j*. Its input converges to a steady-state value *u*_*ss*_, and since the RFMIO is contractive^36^, its density converges to a steady-state that is identical to the steady-state of an RFMIO with the constant input *u*(*t*) ≡ *u*_*ss*_ (see e.g.^54,55^). We can now determine the steady-state values in the consecutive RFMIOs and so on. The next example demonstrates this for a simple network.

### Example 6.

Consider the network in Example 2 that includes *m* = 2 RFMIOs both with dimension *n* = 3. The first RFMIO has rates [*λ*_0_,*λ*_1_,*λ*_2_,*λ*_3_] = [1, 1, 1/4, 1] and input *u*(*t*) = 1. The corresponding Jacobi matrix is

$$[\begin{array}{ccccc}0 & 1 & 0 & 0 & 0\\ 1 & 0 & 1 & 0 & 0\\ 0 & 1 & 0 & 2 & 0\\ 0 & 0 & 2 & 0 & 1\\ 0 & 0 & 0 & 1 & 0\end{array}]\mathrm{.}$$

The Perron eigenvalue [eigenvector] of this matrix is $\sigma =\sqrt{6}$ $[\zeta =[1/2,\sqrt{3/2},5/2,\sqrt{6},1]^{\prime} ]$, and using Thm. 1, we conclude that the steady-state density in RFMIO 1 is $e=[5/6\,4/5\,1/6]^{\prime}$ (compare with Fig. 5), and the steady-state output is *y*_*ss*_ = *σ*^−2^ = 1/6. We can now analyze the second RFMIO. Its rates are all one and the input is the output of RFMIO 1, so at steady-state the rates are $[1/6\,1\,1\,1]^{\prime}$. The corresponding Jacobi matrix is

$$[\begin{array}{ccccc}0 & \sqrt{6} & 0 & 0 & 0\\ \sqrt{6} & 0 & 1 & 0 & 0\\ 0 & 1 & 0 & 1 & 0\\ 0 & 0 & 1 & 0 & 1\\ 0 & 0 & 0 & 1 & 0\end{array}]\mathrm{.}$$

The Perron eigenvalue [eigenvector] of this matrix is *σ* = 2.6819 $[\zeta =[12.7192\,13.926\,6.1926\,2.6819\,1]^{\prime} ]$, and using Thm. 1, we conclude that the steady-state density in RFMIO 2 is $e=[0.1658\,0.1615\,0.139]^{\prime}$ (compare with Fig. 5), and the steady-state output is *y*_*ss*_ = *σ*^−2^ = 0.139.$\square$

Now that we have considered networks with and without feedback connections, we are ready to demonstrate how Problem 1 can be efficiently solved. For a feed-forward network, Prop. 2 implies that the objective function of Problem 1 is strictly concave. For a network with feedback connections, Prop. 1 implies that Problem 1 can be reparametrized so that it becomes strictly concave. The first [second] constraint in (6) is convex [affine] and this implies the following result (see e.g.^56^).

### **Theorem 3**.

*Problem 1 can always be cast as a strictly convex optimization problem*.

Thm. 3 implies in particular that the optimal solution is unique. Moreover, there exist highly efficient numerical algorithms for computing the unique solution even for very large networks.

## A Biological Example

In order to demonstrate how our model can be used to address questions arising in synthetic biology, we consider the problem of maintaining high growth rates for both a highly expressed heterologous protein and a highly expressed endogenous protein in the cell. These issues are currently attracting considerable interest as lack of understanding of the burden of expressing additional genes affects our ability to predictively engineer cells (see e.g.^57^ and the references therein).

Specifically, we consider the problem of maximizing the sum of the steady-state production rates of both heterologous and endogenous genes, under the assumption that they share a common ribosomal resource. We assume that the coding regions of the endogenous gene (which may include various regulatory signals), and the coding region of the heterologous gene (which is optimized to include the most efficient codons) cannot be modified. However, it is possible to engineer the UTRs of these genes in order to modulate their initiation rates. We demonstrate how this biological problem can be modeled and analyzed in the framework of our model.

The endogenous gene is the highly expressed *S*. *cerevisiae* gene YGR192C that encodes the protein TDH3, which is involved in glycolysis and gluconeogenesis. The heterologous gene is the green fluorescent protein (GFP) gene (the GFP protein sequence is from gi:1543069), optimized for yeast (i.e. its codons composition was synonymously modified to consist of optimized yeast codons). The YGR192C gene ORF consists of 332 codons, and the GFP gene ORF of 239 codons. The simultaneous translation of these two genes while using a shared resource is modeled as depicted in Fig. 9, where RFMIO 1 (fed by an input *u* = *v*) models the translation of the YGR192C gene, and RFMIO 2 (fed by the input *u* = 1 − *v*) models the translation of the GFP gene. Here *v* ∈ [0, 1] is a parameter that determines the relative amount of ribosomal resources allocated to each gene.

Similarly to the approach used in^30^, we divide the YGR192C [GFP] mRNA sequnce into 33 [$23$] consecutive subsequences: the first subsequence includes the first nine codons (that are also related to later stages of initiation^58^). The other subsequences include 10 non-overlapping codons each, except for the last subsequence in the YGR192C gene that includes 13 codons. This partitioning was found to optimize the correlation between the RFMIO predictions and biological data.

We model the translation of the YGR192C [GFP] gene using an RFMIO with *n* = 32 [*n* = 23] sites. To determine the RFMIO paramteres we first estimate the elongation rates *λ*_1_, …, *λ*_*n*_, using ribo-seq data for the codon decoding rates^59^, normalized so that the median elongation rate of all *S*. *cerevisiae* mRNAs becomes 6.4 codons per second ^60^. The site rate is (site time)^−1^, where site time is the sum over the decoding times of all the codons in this site. These rates thus depend on various factors including availability of tRNA molecules, amino acids, Aminoacyl tRNA synthetase activity and concentration, and local mRNA folding^1,58,59^.

The initiation rate (that corresponds to the first subsequence) for the YGR192C gene is estimated based on the ribosome density per mRNA levels, as this value is expected to be approximately proportional to the initiation rate when initiation is rate limiting^30,61^. Again, we applied a normalization that brings the median initiation rate of all *S*. *cerevisiae* mRNAs to 0.8 mRNAs per second^62^, and this results in an initiation rate of 2.1958 for the YGR192C gene. The GFP initiation rate was set to 0.8. A calculation shows that when each gene is modeled separately using an RFMIO with *u* = 1, the steady-state production rate of the gene YGR192C [GFP] is *r*_*ss*_ = 0.1859 [*r*_*ss*_ = 0.1892].

**Figure 11:**
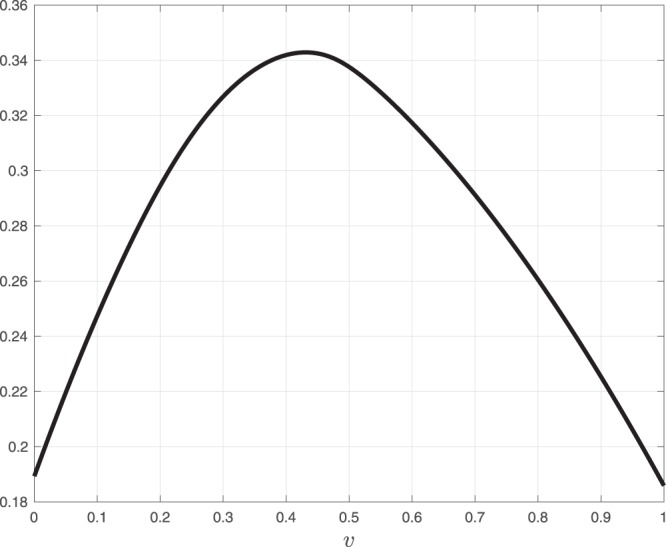
Steady-state network output *y*_*ss*_(*v*) for the of simultaneous translation of YGR192C (RFMIO 1) and GFP (RFMIO 2) genes, using the network depicted in Fig. 9, as a function of the control parameter *v*.

Figure 11 depicts the network output *y*_*ss*_(*v*), as a function of *v* ∈ [0, 1]. The unique maximum *y*_*ss*_(v*) = 0.3429 is attained for v* = 0.4311, which corresponds to feeding a smaller [larger] part of the common ribosomal resource to the GFP [YGR192C] gene. This is reasonable, as the steady-state production rate of the GFP gene is slightly larger than the steady-state production rate of the YGR192C gene. This result implies that in order to maximize the sum of the steady-state production rates of the YGR192C gene and the GFP gene, using a common ribosomal resource, their UTRs binding efficiency should be engineered such that 43% of the ribosomal resource initiates the YGR192C mRNAs, and the remaining 57% initiates the GFP mRNAs. Our analytical approach can also be used to determine the ribosomal allocation that maximizes some weighted sum of the two production rates.

## Discussion

Studying the flow of biological “machines” like ribosomes, RNAPs, or motor proteins along biological networks like interconnected mRNA molecules or filaments is of paramount importance. These biological machines have volume and thus satisfy a simple exclusion principle: two machines cannot be in the same place at the same time.

In order to better understand these cellular biological processes it is important to study the flow of biological “machines” along networks of interconnected “roads”, and not only in isolated processes. We propose to model and analyze such phenomena using networks of interconnected RFMIOs. The RFMIO dynamics satisfies a “soft” simple exclusion principle: as a site becomes fuller the effective entry rate into this site decreases. In particular, “traffic jams” may evolve behind slowly moving machines. The input and output of every RFMIO facilitate their integration into interconnected networks that can be represented using a graph. The nodes in this graph represent the different RFMIOs, and the (weighted) edges describe how the output of each RFMIO is divided between the inputs of other (or the same) RFMIO. Our main result shows that under quite general positive feedback connections, such a network always admits a unique steady-state and any trajectory of the system converges to this steady-state. This opens the door to many interesting research questions, e.g., how does this steady-state depends on the various parameters in the network, and for what feedback connections is the steady-state output of the network optimized?

We demonstrated using various examples how such networks can model interesting biological phenomena like competition for shared resources, the optimal distribution of a shared biological resource between several “clients”, optimizing the effect of ribosome recycling, and more.

The RFM is amenable to rigorous analysis, even when the rates are not homogeneous, using various tools from systems and control theory like contraction theory^63,64^, the theory of cooperative dynamical systems^24,48,65,66^, convex analysis and more. This amenability to rigorous analysis carries over to networks of RFMIOs. For example, the problem of how to connect the RFMIO outputs in the network to inputs so that the steady-state network output is maximized can be cast as a convex optimization problem. This means that the problem can be solved efficiently even for large networks (see, e.g.^56^).

The networks we propose here allow modeling complex biological processes in a coherent and useful manner. The network models static connections between the RFMIOs. The dynamical part is described by the set of ODEs for each RFMIO. The parameters used in these models can be inferred based on various sources of large-scale genomic data (see, for example^59,67^) and/or can be predicted directly from the nucleotide sequence of the gene (see, for example^53,68^). In addition, the analyzed network can be built gradually, one module after another. For example, one can engineer and study one metabolic pathway and then connect another pathway to the existing module (see Fig. 8), etc. This yields a combined model that describes both biophysical aspects of gene expression regulation (e.g. translation), and properties of metabolism (e.g. stoichiometry of enzymes and metabolites, and rates of metabolic reactions).

Topics for further research include networks where the weighted connections between the RFMIOs may also change with time. This may model for example mRNA molecules that diffuse through the cell and consequently change their interactions with ribosomes, other mRNAs, etc. (see. e.g.^69^).

Finally, networks of interconnected TASEPs have been used to model other natural and artificial phenomena such as vehicular traffic and evacuation dynamics. We believe that the deterministic networks proposed here can also be applied to model and analyze such phenomena.

## Supplementary information

Supplementary information
