# Supplementary material for: Networks of ribosome flow models for modeling and analyzing intracellular traffic
Source: Sci Rep. 2019 Feb 8;9:1703. doi: 10.1038/s41598-018-37864-1 (PMC6368613; doi:10.1038/s41598-018-37864-1)
Supplement: Supplementary file 1 — Supplementary information [file 41598_2018_37864_MOESM1_ESM.pdf]

Appendix: Networks of ribosome flow models for modeling and analyzing intracellular traffic

Itzik Nanikashvili, Yoram Zarai, Alexander Ovseevich, Tamir Tuller and Michael Margaliot

PROOFS

The proof of Thm. 2 is based on showing that the network of RFMIOs is a cooperative dynamical system [3] whose trajectories evolve on a compact state-space and with a *unique* equilibrium point in this state-space (in this Appendix we use the term equilibrium point instead of steady-state). We require the following auxiliary result.

Theorem 1. *Consider a network of m RFMIOs in the form:*

$$\begin{aligned} \dot{x}^1 &= f^1(x^1,u^1), & y^1 &= \lambda^1_{n^1} x^1_{n^1},\\ &\vdots \\ \dot{x}^m &= f^1(x^m,u^m), & y^m &= \lambda^m_{n^m} x^m_{n^m}, \end{aligned} \tag{A.1}$$

with the inputs given by

$$u^i = c^i_0 + \sum_{k=1}^{m} c^i_k y^k, \quad i = 1,\dots,m, \tag{A.2}$$

where $c^i_0 > 0$, and $c^i_k \geq 0$ for $k = 1,\dots,m$. This network admits no more than a single equilibrium point in the state-space $(0,1)^{n^1} \times \cdots \times (0,1)^{n^m}$.

Note that this represents a quite general network as the input to every RFMIO may include a contribution from the output of *every* RFMIO in the network, with nonnegative weights. The technical condition $c^i_0 > 0$ is needed to guarantee that $u^i(t) > 0$ for all t and, in particular, $u^i_{ss} > 0$.

Proof of Theorem 1. We begin by considering the case $m = 1$. In this case, the dynamics is

$$\begin{aligned} \dot{x}_1 &= \lambda_0(c_0 + c_1\lambda_n x_n)(1-x_1) - \lambda_1 x_1(1-x_2),\\ \dot{x}_2 &= \lambda_1 x_1(1-x_2) - \lambda_2 x_2(1-x_3),\\ &\vdots \\ \dot{x}_n &= \lambda_{n-1}x_{n-1}(1-x_n) - \lambda_n x_n, \end{aligned} \tag{A.3}$$

I. Nanikashvili is with the School of Electrical Engineering, Tel-Aviv University, Tel-Aviv 69978, Israel. E-mail: itzhakna@gmail.com
Y. Zarai is with the Dept. of Biomedical Engineering, Tel-Aviv University, Tel-Aviv 69978, Israel. E-mail: yoramzar@mail.tau.ac.il
A. Ovseevich is with the Ishlinsky Institute for Problems in Mechanics, Russian Academy of Sciences, pr. Vernadskogo, 101, 119526 Moscow, Russia. E-mail: ovseev@ipmnet.ru
T. Tuller is with the Dept. of Biomedical Engineering and the Sagol School of Neuroscience, Tel-Aviv University, Tel-Aviv 69978, Israel. E-mail: tamirtul@post.tau.ac.il
M. Margaliot is with the School of Electrical Engineering and the Sagol School of Neuroscience, Tel-Aviv University, Tel-Aviv 69978, Israel. E-mail: michaelm@eng.tau.ac.il

with $c_0 > 0$, and $c_1 \geq 0$. Suppose that $e \in (0,1)^n$ is an equilibrium point. Eq. (A.3) yields

$$\begin{aligned} \lambda_0(c_0 + c_1\lambda_n e_n)(1-e_1) &= \lambda_1 e_1(1-e_2) \\ &= \lambda_2 e_2(1-e_3) \\ &\vdots \\ &= \lambda_{n-1}e_{n-1}(1-e_n) \\ &= \lambda_n e_n. \end{aligned} \tag{A.4}$$

It follows that e_n uniquely determines e_{n-1}. Then e_n, e_{n-1} uniquely determine e_{n-2}, and so on. We conclude that e_n uniquely determines e. Suppose that $\tilde{e}$, with $\tilde{e} \neq e$, is another equilibrium point in $(0,1)^n$. Then $\tilde{e}_n \neq e_n$, and we may assume that

$$\tilde{e}_n < e_n. \tag{A.5}$$

Eq. (A.3) yields

$$\lambda_{n-1}\tilde{e}_{n-1}(1-\tilde{e}_n) = \lambda_n\tilde{e}_n < \lambda_n e_n = \lambda_{n-1}e_{n-1}(1-e_n),$$

so

$$\tilde{e}_{n-1} < e_{n-1}.$$

Continuing in this fashion yields

$$\tilde{e}_i < e_i, \quad i = 1, \dots, n. \tag{A.6}$$

On the other-hand, (A.4) yields

$$\begin{aligned} e_1 - \tilde{e}_1 &= \frac{\lambda_n\tilde{e}_n}{\lambda_0(c_0 + c_1\lambda_n\tilde{e}_n)} - \frac{\lambda_n e_n}{\lambda_0(c_0 + c_1\lambda_n e_n)} \\ &= \frac{\lambda_n c_0(\tilde{e}_n - e_n)}{\lambda_0(c_0 + c_1\lambda_n\tilde{e}_n)(c_0 + c_1\lambda_n e_n)} \\ &< 0. \end{aligned}$$

This contradicts (A.6), so we conclude that when $m = 1$ the network admits no more than a single equilibrium.

We now consider the case $m > 1$. Suppose that $e \in (0,1)^{n^1} \times \cdots \times (0,1)^{n^m}$ is an equilibrium point. Write $e = \begin{bmatrix} e^1 \\ \vdots \\ e^m \end{bmatrix}$, where $e^i := \begin{bmatrix} e^i_1 & \dots & e^i_{n^i} \end{bmatrix}'$. For $i = 1, \dots, m$, let $r^i(e) := c^i_0 + \sum_{k=1}^m c^i_k \lambda^k_{n^k} e^k_{n^k}$, i.e. the steady-state input to the ith RFMIO. Then at steady-state

$$\begin{aligned} \lambda^i_0 r^i(e)(1-e^i_1) &= \lambda^i_1 e^i_1(1-e^i_2) \\ &= \lambda^i_2 e^i_2(1-e^i_3) \\ &\vdots \\ &= \lambda^i_{n^i-1}e^i_{n^i-1}(1-e^i_{n^i}) \\ &= \lambda^i_{n^i}e^i_{n^i}. \end{aligned} \tag{A.7}$$

We already know that $e^i_{n^i}$ uniquely determines e^i. Suppose that $\tilde{e} \neq e$ is another equilibrium point of the network. Then $\tilde{e}^i_{n^i} \neq e^i_{n^i}$ for some i. We may assume that $\tilde{e}^1_{n^1} < e^1_{n^1}$. Arguing as in the case $m = 1$ above yields

$$\tilde{e}^1_i < e^1_i, \quad i = 1, \dots, n^1. \tag{A.8}$$

On the other-hand, (A.7) yields

$$\begin{aligned} e_1^1 - \tilde{e}_1^1 &= \frac{\lambda_{n^1}^1 \tilde{e}_{n^1}^1}{\lambda_0^1 r^1(\tilde{e})} - \frac{\lambda_{n^1}^1 e_{n^1}^1}{\lambda_0^1 r^1(e)} \\ &= \lambda_{n^1}^1 \frac{c_0^1(\tilde{e}_{n^1}^1 - e_{n^1}^1) + \sum_{k=2}^m c_k^1 \lambda_{n^k}^k (\tilde{e}_{n^1}^1 e_{n^k}^k - e_{n^1}^1 \tilde{e}_{n^k}^k)}{\lambda_0^1 r^1(\tilde{e}) r^1(e)}. \end{aligned}$$

Combining this with (A.8) implies that at least one of the terms in the summation on the right-hand side must be positive. We may assume that

$$\tilde{e}_{n^1}^1 e_{n^2}^2 > e_{n^1}^1 \tilde{e}_{n^2}^2. \tag{A.9}$$

Thus, $\frac{e_{n^2}^2}{\tilde{e}_{n^2}^2} > \frac{e_{n^1}^1}{\tilde{e}_{n^1}^1} > 1$, and we conclude that

$$\tilde{e}_i^2 < e_i^2, \quad i = 1, \dots, n^2. \tag{A.10}$$

Now (A.7) yields

$$\begin{aligned} e_1^2 - \tilde{e}_1^2 &= \frac{\lambda_{n^2}^2 \tilde{e}_{n^2}^2}{\lambda_0^2 r^2(\tilde{e})} - \frac{\lambda_{n^2}^2 e_{n^2}^2}{\lambda_0^2 r^2(e)} \\ &= \lambda_{n^2}^2 \frac{c_0^2(\tilde{e}_{n^2}^2 - e_{n^2}^2) + c_1^2 \lambda_{n^1}^1 (\tilde{e}_{n^2}^2 e_{n^1}^1 - e_{n^2}^2 \tilde{e}_{n^1}^1) + \sum_{k=2}^m c_k^2 \lambda_{n^k}^k (\tilde{e}_{n^2}^2 e_{n^k}^k - e_{n^2}^2 \tilde{e}_{n^k}^k)}{\lambda_0^2 r^2(\tilde{e}) r^2(e)}. \end{aligned}$$

Combining this with (A.9) and (A.10) implies that at least one of the terms in the summation must be positive. We may assume that

$$\tilde{e}_{n^2}^2 e_{n^3}^3 > e_{n^2}^2 \tilde{e}_{n^3}^3,$$

i.e.

$$\frac{e_{n^3}^3}{\tilde{e}_{n^3}^3} > \frac{e_{n^2}^2}{\tilde{e}_{n^2}^2} > 1.$$

Continuing in this manner, we find that

$$\frac{e_{n^m}^m}{\tilde{e}_{n^m}^m} > \dots > \frac{e_{n^2}^2}{\tilde{e}_{n^2}^2} > \frac{e_{n^1}^1}{\tilde{e}_{n^1}^1} > 1, \tag{A.11}$$

and that

$$\tilde{e}_j^k < e_j^k, \quad k = 1, \dots, m, \ j = 1, \dots, n_k. \tag{A.12}$$

Using (A.7) again yields

$$e_1^m - \tilde{e}_1^m = \lambda_{n^m}^m \frac{c_0^m(\tilde{e}_{n^m}^m - e_{n^m}^m) + \sum_{k=1}^{m-1} c_k^m \lambda_{n^k}^k (\tilde{e}_{n^m}^m e_{n^k}^k - e_{n^m}^m \tilde{e}_{n^k}^k)}{\lambda_0^m r^m(\tilde{e}) r^m(e)}.$$

By (A.11) and (A.12), the left-hand side here is positive and the right-hand side is negative. This contradiction completes the proof. □

We can now prove Thm. 2. For a set $\mathcal{W}$ we denote the interior of $\mathcal{W}$ by $\operatorname{int}(\mathcal{W})$.

Proof of Thm. 2. Write the network (A.1) and (A.2) as $\dot{x} = f(x)$, with $x \in \mathbb{R}^{n^1+\dots+n^m}$. Let $J(x) := \frac{\partial}{\partial x} f(x)$ denote the Jacobian of this dynamics. We claim that $J(x)$ is Metzler for all $x \in \Theta := [0,1]^{n^1} \times \dots \times [0,1]^{n^m}$. Indeed, every RFMIO is a cooperative system, so it has a Metzler Jacobian, and the other non-zero off-diagonal terms in J are due to the connections (A.2) and are nonnegative, as all the c_k^is are nonnegative. We conclude that the network is a cooperative system. It is not difficult to show that Θ is an invariant set, and since it is convex and compact it admits at least one equilibrium point e. Furthermore, it can be shown that for any initial condition $a \in \Theta$ the solution satisfies $x(t, a) \in \operatorname{int}(\Theta)$ for all $t > 0$.

Thus, any equilibrium point satisfies $e \in \text{int}(\Theta)$. By Thm. 1 the network admits a unique equilibrium point e. Now Ji-Fa's Theorem [1] implies that e is GAS. $\square$

Proof of Prop. 1. For the sake of simplicity, we detail the proof for the network with $m = 2$ RFMIOs (the proof in the general case is very similar). In this case, the inputs to the two RFMIOs are

$$u^1(t) = c_0^1 y^0 + c_1^1 y^1(t) + c_2^1 y^2(t),$$
$$u^2(t) = c_0^2 y^0 + c_1^2 y^1(t) + c_2^2 y^2(t),$$

where $y^0 \geq 0$ represents a constant source (e.g. a pool of ribosomes), $c_k^1, c_k^2 \geq 0$ for $k = 0, 1, 2$, and $c_i^1 + c_i^2 \leq 1$ for $i = 0, 1, 2$. Let

$$w_i^k := c_i^k y^i. \tag{A.13}$$

Then

$$u^1 = w_0^1 + w_1^1 + w_2^1,$$
$$u^2 = w_0^2 + w_1^2 + w_2^2, \tag{A.14}$$

and the constraints become

$$w_j^k \geq 0, w_i^1 + w_i^2 \leq y^i. \tag{A.15}$$

We know that the network of RFMIOs converges to a steady-state, and that the steady-state output y_{ss}^i, $i = 1, 2$, is a strictly concave function of the rates in RFMIO i. In particular, $y_{ss}^i = p_i(u_{ss}^i)$, for some strictly concave function p_i. Thus, the steady-state network output is

$$\begin{aligned} y_{ss} &= \sum_{j=1}^{m} v_{j,m+1} y_{ss}^j \\ &= \sum_{j=1}^{m} v_{j,m+1} p_j((w_0^j + w_1^j + w_2^j)_{ss}) \end{aligned} \tag{A.16}$$

This shows that y_{ss} is strictly concave in the steady-state w_k^is. At steady-state, the constraints (A.15) become

$$(w_j^k) \geq 0, w_i^1 + w_i^2 \leq p_i(w_0^i + w_1^i + w_2^i). \tag{A.17}$$

These constraints define a convex set of admissible w_k^is. We conclude that the problem of maximizing (A.16) subject to (A.17) is a convex optimization problem. Determining the optimal w_k^is is thus numerically tractable even for large networks. Once these values are known, we can compute: (1) the optimal steady-state u^is from (A.14); (2) the optimal steady-state outputs y^i (e.g. using the spectral representation); and finally (3) the optimal weights c_i^k from (A.13). $\square$

Proof of Prop. 2 . In a feed-forward network with m RFMIOs, the RFMIOs can be divided into w disjoint sets in the following manner. Let $\mathcal{O}_1 \subset \{1, \dots, m\}$ denote the subset of RFMIOs that are fed only from constant sources. Similarly, let $\mathcal{O}_2 \subset \{1, \dots, m\} \setminus \mathcal{O}_1$ denote the subset of RFMIOs that are fed from the outputs of RFMIOs in $\mathcal{O}_1$ and/or from constant sources, but that are not in $\mathcal{O}_1$, and so on. Note that for $i \neq j$, $\mathcal{O}_i \cap \mathcal{O}_j = \emptyset$, and that $\mathcal{O}_1 \cup \dots \cup \mathcal{O}_w = \{1, \dots, m\}$. It has been shown in [2] that in the RFM the mapping $(\lambda_0, \dots, \lambda_n) \to r_{ss}$ is strictly concave over $\mathbb{R}_{++}^{n+1}$. In particular, the mapping from λ_0 to r_{ss} is strictly concave. This implies that in an RFMIO with a positive constant input $u(t) \equiv v$ the mapping $v \to y_{ss}$ is strictly concave. Consider a feed-forward network of RFMIOs. Pick an RFMIO in $\mathcal{O}_1$. The input to this RFMIO has the form $v^1 u^1 + \dots + v^p u^p$, where the u^is are positive sources and the v^is are control weights. It follows that the mapping $(v^1, \dots, v^p) \to y_{ss}$ of this RFMIO is strictly concave. We

conclude that any weighted sum of outputs of RFMIOs in $\mathcal{O}_1$ is strictly concave in the (relevant) control weights. We can now proceed to RFMIOs in $\mathcal{O}_2$, and so on. $\square$

REFERENCES

[1] J. Ji-Fa, "On the global stability of cooperative systems," *Bull. London Math. Soc.*, vol. 26, pp. 455–458, 1994.

[2] G. Poker, Y. Zarai, M. Margaliot, and T. Tuller, "Maximizing protein translation rate in the nonhomogeneous ribosome flow model: A convex optimization approach," *J. Royal Society Interface*, vol. 11, no. 100, p. 20140713, 2014.

[3] H. L. Smith, *Monotone Dynamical Systems: An Introduction to the Theory of Competitive and Cooperative Systems*, ser. Mathematical Surveys and Monographs. Providence, RI: Amer. Math. Soc., 1995, vol. 41.
